# Comprehensive Screening of Genetic Variants in the Coding Region of *F8* in Severe Hemophilia A Reveals a Relationship with Disease Severity in a Colombian Cohort

**DOI:** 10.3390/life14081041

**Published:** 2024-08-21

**Authors:** Samuel Sarmiento Doncel, Ronald Guillermo Peláez, Pablo Lapunzina, Fernando F. Corrales-Medina, Gina Alejandra Díaz Mosquera, Santiago Bonanad, Javier Mauricio Cortes, Mario Cazalla, Natalia Gallego, Felipe Querol-Giner, Jair Tenorio, José A. López Guerrero

**Affiliations:** 1 Integral Solutions SD SAS, Integral Solutions Research, Bogota 110121, Colombia; 2 Life Sciences and Health Research Group, Graduates School, CES University, Medellin 050021, Colombia; 3 Doctoral School, Catholic University of Valencia San Vicente Mártir (UCV), 46002 Valencia, Spain; 4 Instituto de Genética Médica y Molecular (INGEMM), IdiPaz, Hospital Universitario La Paz, 28046 Madrid, Spain; 5 CIBERER, Centro de Investigación en Red de Enfermedades Raras, Instituto de Salud Carlos III, 28029 Madrid, Spain; 6 ITHACA, European Reference Network on Rare Congenital Malformations and Rare Intellectual Disability, 75019 Paris, France; 7 Division of Pediatric Hematology-Oncology, Department of Pediatrics, University of Miami-Miller School of Medicine, Miami, FL 33136, USA; 8 University of Miami-Hemophilia Treatment Center, Miami, FL 33136, USA; 9 Hospital Universitario y Politécnico La Fe, 46026 Valencia, Spain; 10 Physiotherapy in Motion Multispeciality Research Group (PTinMOTION), Department of Physiotherapy, University of Valencia, 46010 Valencia, Spain; 11 BITGENETIC LAB, Qube Technology Park, Tres Cantos, 28460 Madrid, Spain; 12 Department of Pathology, Medical School, Catholic University of Valencia, San Vicente Martir, 46010 Valencia, Spain; 13 Laboratory of Molecular Biology, Fundación Instituto Valenciano de Oncología, 46009 Valencia, Spain

**Keywords:** hemophilia A, FVIII, mutation, inhibitors, variants, whole sequence exome, pathogenic, *F8*

## Abstract

Hemophilia A is an X-linked disorder characterized by quantitative deficiency of coagulation factor VIII (FVIII) caused by pathogenic variants in the factor 8 (*F8*) gene. Our study’s primary objective was to identify genetic variants within the exonic region of *F8* in 50 Colombian male participants with severe hemophilia A (HA). Whole-exome sequencing and bioinformatics analyses were performed, and bivariate analysis was used to evaluate the relationship between identified variants, disease severity, and inhibitor risk formation. Out of the 50 participants, 21 were found to have 17 different pathogenic *F8* variants (var). It was found that 70% (var = 12) of them were premature truncation variants (nonsense, frameshift), 17.6% (var = 3) were missense mutations, and 11.7% (var = 2) were splice-site variants. Interestingly, 35% (var = 6) of the identified variants have not been previously reported in the literature. All patients with a history of positive inhibitors (n = 4) were found to have high-impact genetic variants (nonsense and frameshift). When investigating the relationship between variant location (heavy versus light chain) and specific inhibitor risk, 75% (n = 3) of the inhibitor participants were found to have variants located in the *F8* light chain (*p* = 0.075), suggesting that conserved domains are associated with higher inhibitor risk. In summary, we identified genetic variants within the *F8* that can possibly influence inhibitor development in Colombian patients with severe HA. Our results provide a basis for future studies and the development of further personalized treatment strategies in this population.

## 1. Introduction

Hemophilia A (HA) is an inherited bleeding disorder characterized by quantitative deficiency of coagulation factor VIII (FVIII) and caused by pathogenic variants in the factor 8 (*F8)* gene located on the X chromosome (Xq28) [1,2,3,4]. The *F8* gene has 26 exons and 187,000 base pairs (bp), and encodes a high-molecular-weight glycoprotein that is 2351 amino acids (aa) long [3,5,6]. Mature FVIII protein consists of six domains arranged in the following order: (A1-A2-B)–(A3-C1-C2) from the amino terminus to the carboxyl terminus [5]. The first domain is known as “heavy chain” and the second as “light chain”. Currently, there are more than 3,756 reported mutation variants within the *F8* gene known to be associated with HA [7,8,9].

Hemophilia A is estimated to affect one in 5000–10,000 live male births [5,10]. According to the 2023 Colombian national registry, developed by the Colombian Fund for High-Cost Diseases (CAC), the incidence and prevalence of HA in Colombia were 0.83 and 4.68 for every 10,000 people, respectively. Out of 2421 reported patients with HA, 52.78% of them were categorized as having a severe disease [11].

Standard treatment for severe HA (residual FVIII level less than 1%) patients includes the use of FVIII concentrates, with the primary goal of achieving a minimum plasma concentration greater than or equal to 1% between FVIII scheduled infusions [12]. The efficacy of replacement-based therapies can be affected by the development of immunoglobulin G (IgG) FVIII-neutralizing alloantibodies. These inhibitors can develop in up to 25–30% of patients with severe HA. The presence of inhibitors makes treatment with FVIII concentrates ineffective, increases the incidence of bleeding events, and affects the overall quality of life of HA patients [1,13]. Inhibitors are classified based on their peak inhibitory titers, with those with historic levels < 5 Bethesda units (BU) classified as low-responding (LR) inhibitors and those with ≥ 5 BU as high-responding (HR) inhibitors. As of 2023, only 6% of HA Colombian patients were reported as having positive inhibitor titers, 50% of them being LR cases [11].

Given the genetic nature of HA, identifying *F8* variants could allow for the detection of possible variants that may be associated with the severity of the condition and inhibitor formation [14]. Prior studies have usually focused on *F8* mutations localized within intron 22 and intron 1, as they account for approximately 45% and 9% of HA cases, respectively, and are the most common mutations reported in HA patients [15]. Considering that the *F8* gene-coding region could have a significant number of other mutations, this study aimed to identify variants in the exonic region of *F8* in a cohort of Colombian patients with severe HA using whole-exome sequencing (WES), and to examine their relationship with disease severity and risk for inhibitor formation.

## 2. Materials and Methods

### 2.1. Study Design

This cross-sectional study was performed via open invitation to 50 male participants with severe HA (FVIII concentration < 1%) affiliated with Integral Solutions SD SAS, a specialized medical care center in Colombia. The study was approved by the Institutional Human Research Ethics Committee at CES University (Project ID: 1065).

### 2.2. Sample Collection and Sequencing

**Figure 1:**
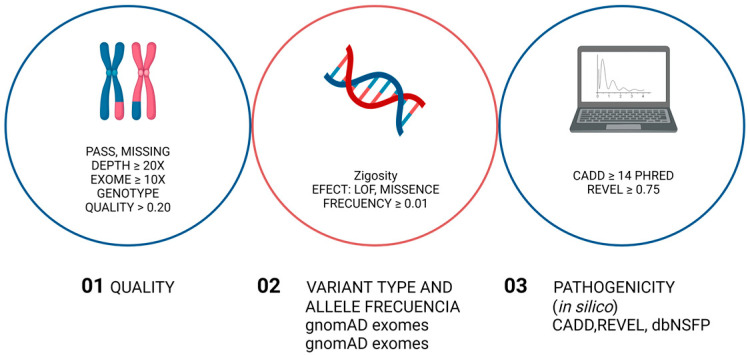
Workflow for variant prioritization of single nucleotide variants and small insertion/deletion variants. The filtering algorithm strategy used in Varseq 2.3.0 for prioritizing variants in *F8* and co-expression-associated genes is shown. This figure was made using BioRender.

A total of 5 mL of peripheral whole blood was collected from each participant. DNA was quantified via spectrophotometry (Nanodrop Lite) by measuring the absorbance at 260 nm, following the manufacturer’s instructions. Library preparation was performed using an Agilent SureSelect M kit (v6.0). The sequencer applied was a NovaSeq6000 platform (Illumina, San Diego, CA, USA), following the manufacturer’s instructions. Variant prioritization was given for the filtering algorithm in Varseq 2.3.0, applying the following strategy: quality (PASS and Missing); depth ≥ 20×; genotype quality ≥ 20.2; type of variant and frequency, filtered via zygosity; effect: (LOF, Missense); and a pseudocontrol population frequency ≤ 1% according to gnomAD exomes, gnomAD genomes, 1000G, ESP, Kaviar, Beacoz, and Bravo. Pathogenicity prediction was performed by filtering using the threshold CADD ≥ 14 phred and Revel ≥ 0.75 to identify variants predicted to be damaging using in silico bioinformatics tools (Figure 1). Pathogenicity assignment for the remaining variants after filtering was performed according to the 2015 American College of Medical Genetics and Genomics (ACMG) recommendations. Only those variants that were likely to be pathogenic and/or variants of unknown significance (VUS) were reported. To analyze copy number variation (CNV), our filtering strategy included deletions, duplications, *p*-value < 0.05, a Span of 10,0000 pb, and clinical interpretations of “likely pathogenic” and “pathogenic”.

### 2.3. Protein Structure Prediction

The canonical amino acid sequence of *F8* was obtained from the UniProt database [16]. Considering this as our base, this sequence was modified according to the amino acid changes in each of the included patients. Once this edit was completed, the FVIII protein with the corresponding changes was modeled using the Swiss-Model Workspace [17]. In total, 15 variants were modeled, and the generated models were downloaded in PDB format in parallel with the download of the PDB file of the wild-type FVIII protein [16,18,19] and uploaded into PyMOL [20] for visualization and editing. Subsequently, visualization of all downloaded predictions was performed, coloring the mutated protein domains with the same range of colors as the wild-type domains.

### 2.4. Statistical Analysis

For univariate (descriptive) analyses, we applied frequency statistics to describe the behavior of each variable to be analyzed. For bivariate analyses, we applied Fisher’s statistical test for small sample sizes. All estimates were performed at a confidence level of 95%. All statistical analyses were performed using SPSS Statistics for Windows version 23.

## 3. Results

### 3.1. F8 Variants Identified in Study Participants

**Figure 2:**
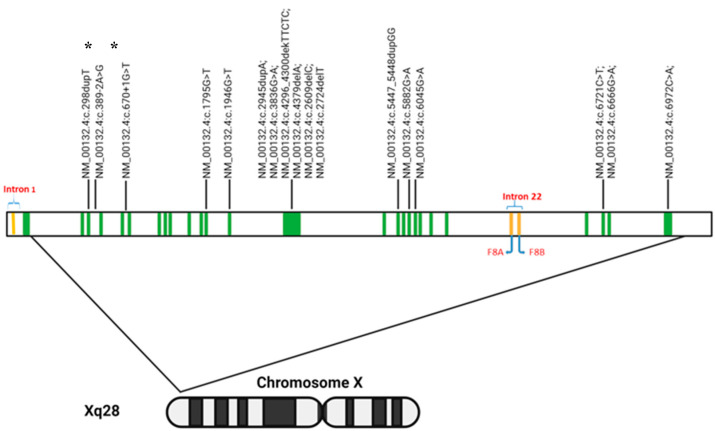
Location of mutations in *F8* variants (variant nomenclature). The *F8* gene contains two regions, *F8A* and *F8B*, in intron 22 that act as a bidirectional promoter, transcribing in the forward and reverse directions. * indicates splice variants.

Whole exome sequencing analysis identified potential pathogenic *F8* variants in 21 of the 50 participants, with a total of 17 different F8 variants (Figure 2). The majority of participants had frameshift variants (52.4%, n = 11), followed by nonsense variants (23.8%, n = 5), missense variants (14.3%, n = 3), and splice-site variants (9.5%, n = 2) (Table 1). Some 57% (n = 12) of participants had variants located within the heavy chain, and 33.3% (n = 7) within the light chain. Two (9.5%) participants presented splicing variants (Table 1 and Table 2). 

Out of the 21 participants with mutations, 16 had mutations resulting in early termination of protein expression. Of these, 5 presented variants generated by the change of a single nitrogenous base, leading to an immediate termination codon (nonsense mutation). Eleven participants presented deletions or duplications of one or more nitrogenous base, which changed the reading frame (frameshift). No CNVs were detected.

Out of the 17 identified *F8* variants, 70% (var = 12) exhibited terminated protein synthesis (nonsense and frameshift mutations), suggesting alterations of the protein, 17.6% (var = 3) were missense mutations, and 11.7% (var = 2) were split-site variants (Table 3). Moreover, 65% (var = 11) of the variants were found within the heavy chain, whereas 35% (var = 6) were located in the light chain.

Variants in the light chain were mainly found in the A3 and C2 domains. Most of these variants exhibited early termination in protein synthesis, destabilizing the overall structure of FVIII, which can lead to a loss of affinity and interaction between FVIII and other hemostatic proteins involved in the coagulation process. This was observed, for example, in the c.6972C>A p.(Tyr2324Ter) variant, which is truncated at the nucleotide positions 2303–2332, representing the carboxyl-terminal sequence and one of the 3 VWF binding regions, which will lead to early FVIII proteolysis [21]. The c.6666G>A p.(Trp2222Ter) and c.6721C>T p.(Gln2241Ter) variants exhibit early termination in the C2 domain, also associated with a decrease in affinity to VWF. These mutations also cause aminophospholipid protein binding sites to lose phosphatidylserine residues on the surface of platelets, affecting platelet packaging [22]. The c.6045G>A p. (Trp2015Ter) and c.5882G> p. (Trp1961Ter) variants present early termination of protein expression in the A3 domain, leading to the loss of FVIII function and loss of multiple sites that contribute to the binding between LRP (high-density lipoprotein receptors) and FVIII [5].) Finally, the c.5447_5448dupGG p. (Gln1817GlyfsTer55) variant is associated with truncating protein synthesis that affects the FVIII region from amino acids 1803–1818, which is critical for the function of the cofactor [23].

**Figure 3:**
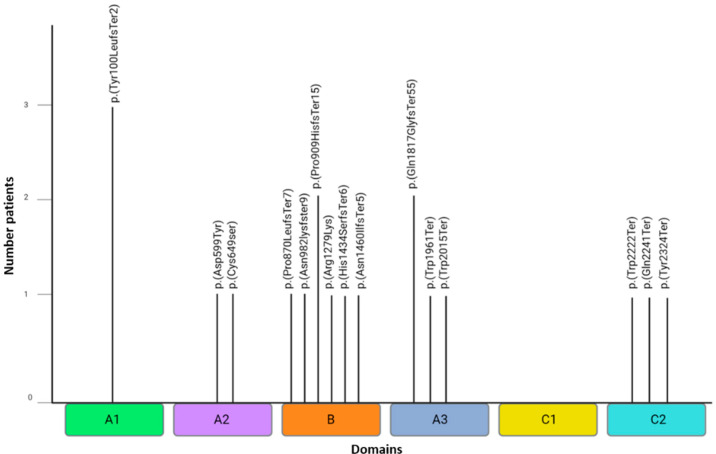
Locations of the protein mutations per domain (protein change nomenclature). This figure shows the location of mutations in the variants of *F8*. The variants c.670+1G>T and c.389-2A>G are not shown because they are splicing variants.

Variants in the heavy chain, such as c.2724delT p.(Pro909HisfsTer15), c.4296_4300delTTCTC p.(His1434SerfsTer6), c.2609delC p.(Pro870LeufsTer7), c.2945dupA p.(Asn982LysfsTer9), and c.4379delA p.(Asn1460IlefsTer5), exhibit early termination of the FVIII B domain which, although cleaved upon FVIII activation, is important in the structure of FVIII to allow appropriate interaction with activated FIX (FIXa) and the tenase complex (X) [23]. Two additional missense variants were identified in the A2 domain: c. 1795G>T p. (Asp599Tyr) and c.1946G>C p. (Cys649Ser). In the A1 domain, the most frequently identified variant was the c.298dupT p. (Tyr100LeufsTer2), which is known to lead to premature termination of FVIII protein expression (Figure 3). 

### 3.2. Relationship between F8 Identified Variants and Inhibitor Risk

Out of the 21 participants, 4 (19%) had a history of FVIII inhibitors, 2 were LR and 2 were HR. Inhibitor development in all 4 participants occurred after exposure to high doses (>80 IU/kg/day, for 3 to 7 days) of plasma-derived F8 concentrates for the control of acute bleeding episodes. All 4 participants exhibited variants that cause early termination of FVII synthesis (Table 4).

High-impact variants (nonsense, frameshift) were found in all 4 patients. No relationship was found between coding impact and inhibitor development (*p* = 0.45) (Table 5). All high-impact variants (nonsense, frameshift) seemed to affect 100% of the patients with inhibitors.

When investigating the relationship between the variant’s location (heavy versus light chain) and their specific inhibitor risk, of the inhibitor participants (n = 4) were found to have variants located within the light chain (*p* = 0.075; Table 6).

### 3.3. Previously Unreported F8 Variants

Our analysis found six variants which were not reported in the CHAMP update 2020 report, VarSome Suite, Franklin Genoox, or NCBI: c.2724delT, c.389-2A>G, c.1795G>T, c.2609delC, c.298dupT, and c.5447_5448dupGG.

The c.298dupT variant was present in three participants. Two of them have received primary prophylaxis for more than 15 years without a history of inhibitors or joint damage. The third participant was an older adult who has previously received tertiary prophylaxis and currently has high-response inhibitors following an on-demand regimen, with no bleeding in the last 12 months. Variants c.389-2A>G, c.1795G, and c.2609delC were all present in one participant, who had no joint damage or inhibitors.

The c.2724delT variant was found in two participants currently on prophylaxis regimen without joint damage or history of inhibitors. Finally, the c.5447_5448dupGG variant was present in two other participants currently receiving tertiary prophylaxis with no significant bleeding phenotype in the last 12 months, although one of them currently has low-response inhibitors. 

### 3.4. Three-Dimensional Structure of the F8 Protein

**Figure 4:**
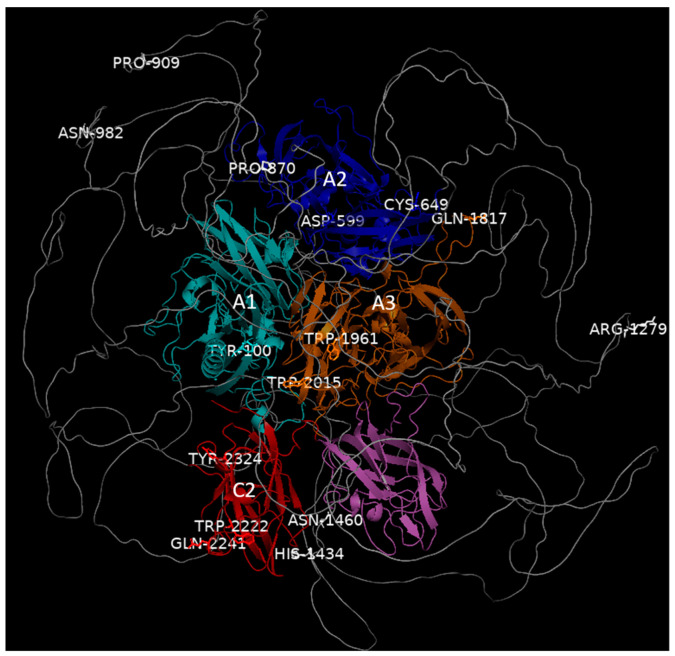
Location of the affected amino acids in the three-dimensional protein structure of FVIII resulting from mutations in the coding region of the *F8* gene. This figure was made by the authors using the PyMOL tool.

**Figure 5:**
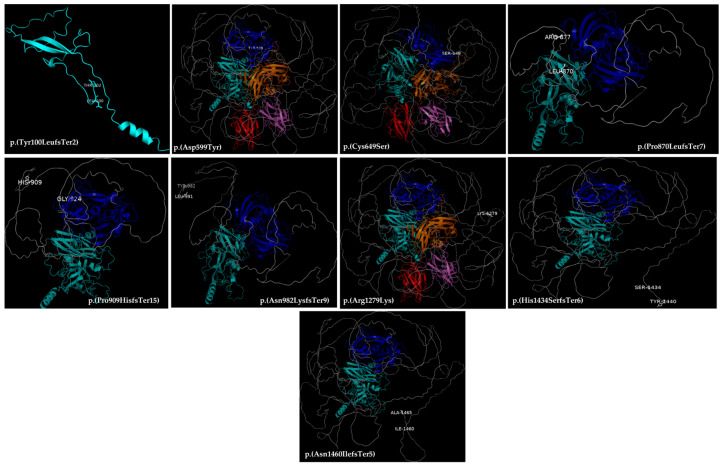
Models of the three-dimensional structure of the FVIII protein. The changes in the amino acids in the heavy chain are highlighted. All models are made by the authors using the PyMOL tool.

**Figure 6:**
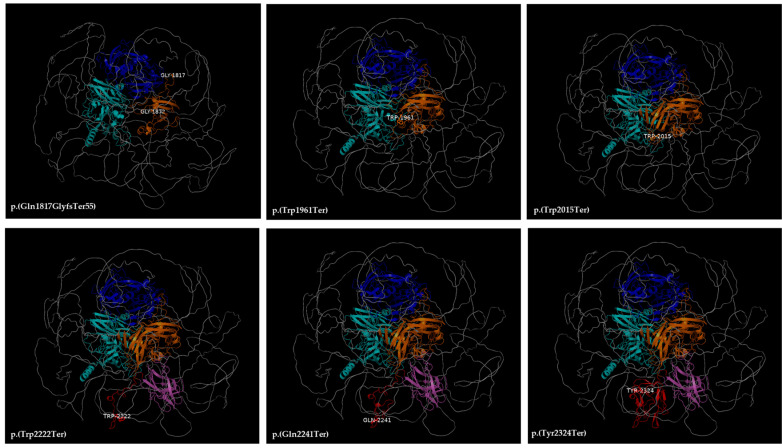
Models of the three-dimensional structure of the FVIII protein. The changes in the amino acids in the light chain are highlighted. All models are made by the authors using the PyMOL tool.

In silico protein modeling allowed us to identify and study the mechanism by which the variants detected in *F8* in our patients affected the structure, localization, and interaction of the FVIII protein and other proteins. Figure 4 shows the 3D structure of the canonical FVIII protein, where the domains are differentiated by color and the affected amino acids are labeled. Figure 5 represents the tertiary structure models of FVIII, showing where the nine variants seem to affect the domains in the heavy chain, which was truncated in most cases. Figure 6 shows the tertiary structure models of FVIII in the light chain, where the protein was truncated in all cases.

## 4. Discussion

Several studies have investigated the role of F8 variants in patients with HA [9,24]. Their findings suggest that the frequency and type of F8 variants, as well as their potential association with inhibitor risk formation, depend on the specific studied population. Due to its known high prevalence, prior HA studies in Colombia have mostly focused on analyzing the role of intron 22 inversion, but have not investigated the prevalence and role of variants located within the exonic region of F8 [15].

In our cohort, and similar to the systematic review by Gouw et al. [25], frameshift type variants were the most common type of identified *F8* variants (52%), followed by nonsense variants, present in 24% of study participants. Interestingly, missense variants were only present in 14% of our cohort. This finding differs from those reported by Gouw and Atik [25,26], where the prevalence of this type of variant ranged between 34% and 45% (Table 7)**.** Nonsense mutations had a similar reported prevalence in comparison to these two studies (24% versus 23% and 25%, respectively). Splice-site variants were more frequently encountered in our cohort.

When looking at variants associated with an early termination of the FVIII protein (frameshift and nonsense mutations), their frequency was higher in our cohort compared to the results reported by Gouw and Atik (76% versus 59% and 50%, respectively). Interestingly, in our cohort, most of the participants (75%) with positive inhibitor history had their variants located in the light chain. This finding also differs from the one reported by Gouw et al. (75% versus 52%, respectively) [8]. We can then hypothesize, and as reported by Oldenburg et al. [27], Carcao and Goudemans [28], and Gensana et al. [29], that the more domains are conserved, the greater the possibility of generating inhibitors. This may be because when more domains are conserved, more epitope sites become available to generate inhibitors, triggering an immune response to exogenous FVIII [30]. 

Another important difference worth highlighting is that, despite IgG epitopes having been most commonly reported to be localized in the A2 and C2 domains where missense variants increase the risk of developing inhibitors up to four-fold [31], in our cohort, variants located in the A2 domain had the lowest prevalence (11.7%, n = 2). These variants were all missense mutations without associated inhibitor risk. This finding could possibly explain the low incidence (5.7%) of patients with hemophilia A and inhibitors reported in the Colombian national registry [11] in comparison to the 25–30% inhibitor rate reported in several other studies [32].

Our study identified six mutations that had not been previously reported in the literature. This finding might suggest that in Colombia, for every ten identified *F8* variants, three might differ from those reported in other populations. This genetic characteristic could possibly explain the different phenotype and inhibitor prevalence observed in Colombian patients with HA. Our findings mandate the need to develop future studies exploring the role of specific treatment regimens based on individual patient genotype, as this strategy might not only lead to improved treatment outcomes but might also impact the cost-effectiveness of these personalized therapies.

Nevertheless, we need to acknowledge that one of the limitations of WES analysis is that it can only detect variants in the exonic region of *F8*. In hemophilia, about 50% of patients have variants in the exonic region, for which the use of other methods that allow for the detection of intron 1 and intron 22 inversions is mandated.

## 5. Conclusions

Our study shows differences in the frequency and type of pathogenic *F8* variants in a cohort of Colombian patients with HA compared to other previously reported HA populations. Furthermore, more than a third of identified *F8* variants have not been previously described in the literature. Interestingly, none of these novel variants were associated with a risk for FVIII inhibitor formation. In our cohort, we also found a greater association of inhibitor presence between *F8* variants located in the FVIII light chain compared to the heavy chain, which could indicate that the more conserved the domains are, the greater the probability of inhibitor generation. Moreover, those variants located in the C2 domain seemed to have the highest risk for inhibitor formation 

Based on our findings, we propose that population-specific F8 genotype information, along with patient bleeding phenotype, can help to develop personalized treatment regimens to further optimize their effectiveness and safety. Population-specific *F8* genotyping can also allow for the identification of HA patients with the highest risk for inhibitor formation. Certainly, these are two important steps towards advancing precision medicine adapted to diverse patient populations. 

## Figures and Tables

**Table 1:** Causative variants identified using whole-exome sequencing in Colombian patients with severe hemophilia A (hg38).

Patient Code	Gene	cDNA Variant NM_00132.4 (*F8*)	Exon	Protein Position	Variant Effect	ACMG Classification	Inhibitors	Affected Protein Domains
ISMG03	*F8*	c.6045G>A	exon 19 of 26 position 47 of 117	p.(Trp2015Ter)	nonsense	Pathogenic	Without inhibitor	-F5/8 type A3(Plastocyanin-like 5) -F5/8 type C1 (complete: 2040-2188) -F5/8 type C2 (complete: 2193-2345)
ISMG04	*F8*	c.5882G>A	exon 18 of 26 position 67 of 183	p.(Trp1961Ter)	nonsense	Pathogenic	High response (8.0)	-F5/8 type A3(Plastocyanin-like 5) -F5/8 type C1 (complete: 2040-2188) -F5/8 type C2 (complete: 2193-2345)
ISMG05	*F8*	c.6972C>A	exon 26 of 26 position 72 of 1961	p.(Tyr2324Ter)	nonsense	Pathogenic	Without inhibitor	-F5/8 type C2 (partial: 2193-2345)
ISMG06	*F8*	c.2724delT	exon 14 of 26 position 611 of 3106	p.(Pro909HisfsTer15)	Frameshift	Pathogenic	Without inhibitor	-B (partial: 760-1667) -F5/8 type A3(Plastocyanin-like 5) -F5/8 type C1 (complete: 2040-2188) -F5/8 type C2 (complete: 2193-2345)
ISMG07	*F8*	c.2724delT	exon 14 of 26 position 611 of 3106	p.(Pro909HisfsTer15)	Frameshift	Pathogenic	Without inhibitor	-B (partial: 760-1667) -F5/8 type A3(Plastocyanin-like 5) -F5/8 type C1 (complete: 2040-2188) -F5/8 type C2 (complete: 2193-2345)
ISMG08	*F8*	c.4296_4300delTTCTC	exon 14 of 26 position 2183-2187 of 3106	p.(His1434SerfsTer6)	Frameshift	Pathogenic	Without inhibitor	-B (partial: 760-1667) -F5/8 type A3(Plastocyanin-like 5) -F5/8 type C1 (complete: 2040-2188) -F5/8 type C2 (complete: 2193-2345)
ISMG09	*F8*	c.5447_5448dupGG	exon 16 of 26 before position 76 of 213	p.(Gln1817GlyfsTer55)	Frameshift	Pathogenic	Without inhibitor	-F5/8 type A3(Plastocyanin-like 5) -F5/8 type C1 (complete: 2040-2188) -F5/8 type C2 (complete: 2193-2345)
ISMG14	*F8*	c.670+1G>T	intron 5 of 25 position 1 of 2433 splicing, intronic)	p.?		Pathogenic	Without inhibitor	
ISMG17	*F8*	c.389-2A>G	intron 3 of 25 position 3823 of 3824 splicing, intronic)	p.?		Pathogenic	Without inhibitor	
ISMG19	*F8*	c.1795G>T	exon 12 of 26 position 43 of 151	p.(Asp599Tyr)	Missense	Likely pathogenic	Without inhibitor	-F5/8 type A 2 (Plastocyanin-like 4)
ISMG21	*F8*	c.3836G>A	exon 14 of 26 position 1723 of 3106	p.(Arg1279Lys)	Missense	VUS	Without inhibitor	-F5/8 type B
ISMG22	*F8*	c.1946G>C	exon 13 of 26 position 43 of 210	p.(Cys649Ser)	Missense	Pathogenic	Without inhibitor	-F5/8 type A 2 (Plastocyanin-like 4)
ISMG23	*F8*	c.6666G>A	exon 24 of 26 position 92 of 149	p.(Trp2222Ter)	Nonsense	Pathogenic	Without inhibitor	-F5/8 type A3(Plastocyanin-like 5) -F5/8 type C1 (complete: 2040-2188) -F5/8 type C2 (complete: 2193-2345)
ISMG25	*F8*	c.6721C>T	exon 24 of 26 position 147 of 149	p.(Gln2241Ter)	Nonsense	Pathogenic	Low response (0.6)	-F5/8 type C 2
ISMG30	*F8*	c.298dupT	exon 3 of 26 before position 34 of 123	p.(Tyr100LeufsTer2)	Frameshift	Pathogenic	Without inhibitor	-F5/8 type A 1 -F5/8 type A 2 -Region B -F5/8 type A 3 -F5/8 type C 1 -F5/8 type C 2
ISMG31	*F8*	c.2609delC	exon 14 of 26 position 496 of 3106	p.(Pro870LeufsTer7)	Frameshift	Pathogenic	Without inhibitor	-B (partial: 760-1667) -F5/8 type A 3 (complete: 1713-2040) -F5/8 type C1 (complete: 2040-2188) -F5/8 type C2 (complete: 2193-2345)
ISMG32	*F8*	c.298dupT	exon 3 of 26 before position 34 of 123	p.(Tyr100LeufsTer2)	Frameshift	Pathogenic	High response (16.0)	-F5/8 type A 1 -F5/8 type A 2 -Region B -F5/8 type A 3 -F5/8 type C 1 -F5/8 type C 2
ISMG34	*F8*	c.2945dupA	exon 14 of 26 before position 833 of 3106	p.(Asn982LysfsTer9)	Frameshift	Pathogenic	Without inhibitor	-B (partial: 760-1667) -F5/8 type A 3 (complete: 1713-2040) -F5/8 type C1 (complete: 2040-2188) -F5/8 type C2 (complete: 2193-2345)
ISMG35	*F8*	c.298dupT	exon 3 of 26 before position 34 of 123	p.(Tyr100LeufsTer2)	Frameshift	Pathogenic	Without inhibitor	-F5/8 type A 1 -F5/8 type A 2 -Region B -F5/8 type A 3 -F5/8 type C 1 -F5/8 type C 2
ISMG38	*F8*	c.5447_5448dupGG	exon 16 of 26 before position 76 of 213	p.(Gln1817GlyfsTer55)	Frameshift	Pathogenic	Low response (1.0)	-F5/8 type A3(Plastocyanin-like 5) -F5/8 type C1 (complete: 2040-2188) -F5/8 type C2 (complete: 2193-2345)
ISMG43	*F8*	c.4379delA	exon 14 of 26 position 2266 of 3106	p.(Asn1460IlefsTer5)	Frameshift	Pathogenic	Without inhibitor	-B (partial: 760-1667) -F5/8 type A 3 (complete: 1713-2040) -F5/8 type C1 (complete: 2040-2188) -F5/8 type C2 (complete: 2193-2345)

**Table 2:** Identified Variants by Type and Location.

			n (%)
Chain	Heavy	A1	3 (14.3%)
		A2	2 (9.5%
		B	7 (33.3%)
	Light	A3	4 (19.0)
		C1	0 (0.0)
		C2	3 (14.3%)
	Splicing		2 (9.5%)
Type of variants	Frameshift		11 (52.4)
	Missense		3 (14.3%)
	Nonsense		5 (23.8%)
	Splicing		2 (9.5%)

**Table 3:** Specific *F8* Variants Identified in the Study Cohort.

Variants	n (%)	Domain	Coding Impact
c.298dupT	3 (14.3%)	A1	Frameshift
c.1795G>T	1 (4.8%)	A2	Missense
c.1946G>C	1 (4.8%)	A2	Missense
c.2724delT	2 (9.5%)	B	Frameshift
c.2609delC	1 (4.8%)	B	Frameshift
c.2945dupA	1 (4.8%)	B	Frameshift
c.3836G>A	1 (4.8%)	B	Missense
c.4296_4300delTTCTC	1 (4.8%)	B	Frameshift
c.4379delA	1 (4.8%)	B	Frameshift
c.5447_5448dupGG	2 (9.5%)	A3	Frameshift
c.5882G>A	1 (4.8%)	A3	Nonsense
c.6045G>A	1 (4.8%)	A3	Nonsense
c.6666G>A	1 (4.8%)	C2	Nonsense
c.6721C>T	1 (4.8%)	C2	Nonsense
c.6972C>A	1 (4.8%)	C2	Nonsense
c.389-2A>G	1 (4.8%)	-	Splicing
c.670+1G>T	1 (4.8%)	-	Splicing
Total	21 (100%)		

**Table 4:** Observed Variants on Patients with Inhibitors.

Inhibitors	Variants (NM_000132.4)	Coding Impact	Chain (Light/Heavy)	Frequency (%)
Without inhibitors	See Table 1			17(81%)
Low response	c.5447_5448dupGG (Exon 16) c.6721C>T (Exon 24)	Frameshift Nonsense	A3 C2	2 (9.5%)
High response	c.5882G>A (Exon 18) c.298dupT (Exon 3)	Nonsense Frameshift	A3 A1	2 (9.5%)
Total				21 (100%)

**Table 5:** Statistical test between coding impact and the presence of inhibitors.

	Inhibitors		
Coding Impact	Yes	No	* *p*-Value
Frameshift	2	9	0.457
Missense	0	3	
Nonsense	2	3	
Splicing	0	2	
Total	4	17	

* Fisher’s exact test.

**Table 6:** Variant Location and Inhibitor Risk.

	Inhibitors		
Chain Location	Yes	No	*p*-Value
Heavy	1	11	0.075
Light	3	4	
Total	4	17	

Fisher’s exact test. Note: additional patients harbored splicing variants in the absence of inhibitors.

**Table 7:** Comparison of the results reported by meta-analyses.

Coding Impact	Cohort (%)	Gouw et al. (%)	Atik et al. (%)
Missense	14	34	45
Nonsense	24	23	25
Frameshift	52	36	25
Splice variant	10	7	5

Data were adapted from a meta-analysis by Gouw et al. [25], and a mutation study by Atik et al. [26], taking only the type of variation found in the study cohort as 100%.

## Data Availability

The data used to develop the article consist of both public and private data. The primary focus was on private and confidential data obtained from patients affiliated with a private health entity. Additionally, publicly available data can be found in the referenced articles, and CHAMP data can be accessed on the following page: https://www.cdc.gov/hemophilia/mutation-project/?CDC_AAref_Val, accessed on 14 August 2024.

## References

[1] Gouw S.C., van den Berg H.M., le Cessie S., van der Bom J.G. (2007). Treatment characteristics and the risk of inhibitor development: A multicenter cohort study among previously untreated patients with severe hemophilia A. J. Thromb. Haemost..

[2] Protocolo-hemofilia-marzo-2015.pdf. https://www.minsalud.gov.co/sites/rid/Lists/BibliotecaDigital/RIDE/DE/CA/Protocolo-hemofilia-marzo-2015.pdf.

[3] Bolton-Maggs P.H.B., Pasi K.J. (2003). Haemophilias A and B. Lancet.

[4] Tantawy A.A.G. (2010). Molecular genetics of hemophilia A: Clinical perspectives. Egypt J. Med. Hum. Genet..

[5] Bogdanova N., Markoff A., Pollmann H., Nowak-Göttl U., Eisert R., Wermes C., Todorova A., Eigel A., Dworniczak B., Horst J. (2005). Spectrum of molecular defects and mutation detection rate in patients with severe hemophilia A. Hum. Mutat..

[6] CDC Centers for Disease Control and Prevention. 2023. CHAMP|Hemophilia|CDC. https://www.cdc.gov/hemophilia/mutation-project/?CDC_AAref_Val=https://www.cdc.gov/ncbddd/hemophilia/champs.html.

[7] Gouw S.C., Van Der Bom J.G., Van Den Berg H.M., Zewald R.A., Ploos Van Amstel J.K., Mauser-Bunschoten E.P. (2011). Influence of the type of F8 gene mutation on inhibitor development in a single centre cohort of severe haemophilia A patients. Haemophilia.

[8] Spena S., Garagiola I., Cannavò A., Mortarino M., Mannucci P.M., Rosendaal F.R., Peyvandi F., El-Beshlawy A., Elalfy M., the SIPPET Study Group (2018). Prediction of factor VIII inhibitor development in the SIPPET cohort by mutational analysis and factor VIII antigen measurement. J. Thromb. Haemost..

[9] Castro H.E., Briceño M.F., Casas C.P., Rueda J.D. (2014). The History and Evolution of the Clinical Effectiveness of Haemophilia Type A Treatment: A Systematic Review. Indian J. Hematol. Blood Transfus..

[10] (2023). Situación de la hemofilia y otras coagulopatías en Colombia 2023—Cuenta de Alto Costo. https://cuentadealtocosto.org/publicaciones/situacion-de-la-hemofilia-y-otras-coagulopatias-en-colombia-2023/.

[11] Nilsson I.M., Berntorp E., Löfqvist T., Pettersson H. (1992). Twenty-five years’ experience of prophylactic treatment in severe haemophilia A and B. J. Intern. Med..

[12] Iorio A., Halimeh S., Holzhauer S., Goldenberg N., Marchesini E., Marcucci M., Young G., Bidlingmaier C., Brandao L.R., Ettingshausen C.E. (2010). Rate of inhibitor development in previously untreated hemophilia A patients treated with plasma-derived or recombinant factor VIII concentrates: A systematic review. J. Thromb. Haemost..

[13] Collins P.W. (2012). Personalized prophylaxis. Haemophilia.

[14] Yunis L.K., Linares A., Cabrera E., Yunis J.J. (2018). Systematic molecular analysis of hemophilia A patients from Colombia. Genet. Mol. Biol..

[15] UniProt: The Universal Protein Knowledgebase in 2023|Nucleic Acids Research|Oxford Academic. https://academic.oup.com/nar/article/51/D1/D523/6835362.

[16] Waterhouse A., Bertoni M., Bienert S., Studer G., Tauriello G., Gumienny R., Heer F.T., de Beer T.A.P., Rempfer C., Bordoli L. (2018). SWISS-MODEL: Homology modelling of protein structures and complexes. Nucleic Acids Res..

[17] Varadi M., Anyango S., Deshpande M., Nair S., Natassia C., Yordanova G., Yuan D., Stroe O., Wood G., Laydon A. (2022). AlphaFold Protein Structure Database: Massively expanding the structural coverage of protein-sequence space with high-accuracy models. Nucleic Acids Res..

[18] Jumper J., Evans R., Pritzel A., Green T., Figurnov M., Ronneberger O., Tunyasuvunakool K., Bates R., Žídek A., Potapenko A. (2021). Highly accurate protein structure prediction with AlphaFold. Nature.

[19] PyMOL | pymol.org. https://pymol.org/.

[20] Nguyen P.-C.T., Lewis K.B., Ettinger R.A., Schuman J.T., Lin J.C., Healey J.F., Meeks S.L., Lollar P., Pratt K.P. (2014). High-resolution mapping of epitopes on the C2 domain of factor VIII by analysis of point mutants using surface plasmon resonance. Blood.

[21] Mannucci P.M., Tuddenham E.G. (2001). The hemophilias--from royal genes to gene therapy. N. Engl. J. Med..

[22] Fay P.J. (2006). Factor VIII Structure and Function. Int. J. Hematol..

[23] Wang J., Yu C., Zhuang J., Qi W., Jiang J., Liu X., Zhao W., Cao Y., Wu H., Qi J. (2022). The role of phosphatidylserine on the membrane in immunity and blood coagulation. Biomark. Res..

[24] Antonarakis S.E., Rossiter J.P., Young M., Horst J., de Moerloose P., Sommer S.S., Ketterling R.P., Kazazian H.H., Negrier C., Vinciguerra C. (1995). Factor VIII gene inversions in severe hemophilia A: Results of an international consortium study. Blood.

[25] Gouw S.C., Berg H.M.v.D., Oldenburg J., Astermark J., de Groot P.G., Margaglione M., Thompson A.R., van Heerde W., Boekhorst J., Miller C.H. (2012). F8 gene mutation type and inhibitor development in patients with severe hemophilia A: Systematic review and meta-analysis. Blood.

[26] Atik T., Işık E., Onay H., Akgün B., Shamsali M., Kavaklo K., Evim M., Tüysüz G., Özbek N.Y., Şahin F. (2020). Factor 8 Gene Mutation Spectrum of 270 Patients with Hemophilia A: Identification of 36 Novel Mutations. Turk J. Haematol..

[27] Oldenburg J., Schröder J., Hermann Brackmann H., Müller-Reible C., Schwaab R., Tuddenham E. (2004). Environmental and genetic factors influencing inhibitor development 1. Semin. Hematol..

[28] Carcao M., Goudemand J. (2018). Los Inhibidores en la Hemofilia: Información Básica.

[29] Gensana M., Altisent C., Aznar J.A., Casaña P., Hernández F., Jorquera J.I., Magallón M., Massot M., Puig L. (2001). Influence of von Willebrand factor on the reactivity of human factor VIII inhibitors with factor VIII. Haemophilia.

[30] Kaveri S.V., Dasgupta S., Andre S., Navarrete A.-M., Repessé Y., Wootla B., Lacroix-Desmazes S. Factor VIII inhibitors: Role of von Willebrand factor on the uptake of factor VIII by dendritic cells. https://onlinelibrary.wiley.com/doi/10.1111/j.1365-2516.2007.01575.x.

[31] Oldenburg J., Pavlova A. (2006). Genetic risk factors for inhibitors to factors VIII and IX. Haemophilia.

[32] Cormier M., Batty P., Tarrant J., Lillicrap D. Advances in Knowledge of Inhibitor Formation in Severe Haemophilia A. https://onlinelibrary.wiley.com/doi/10.1111/bjh.16377.

